# Personality, Perceived Environment, and Behavior Systems Related to Future Smoking Intentions among Youths: An Application of Problem-Behavior Theory in Shanghai, China

**DOI:** 10.1371/journal.pone.0122276

**Published:** 2015-03-31

**Authors:** Yong Cai, Rui Li, Jingfen Zhu, Li Na, Yaping He, Pam Redmon, Yun Qiao, Jin Ma

**Affiliations:** 1 School of Public Health, Shanghai Jiao Tong University, Shanghai, China; 2 Global Health Institute, Emory University, Atlanta, Georgia, United States of America; 3 Pudong Institute for Health Development, Shanghai, China; TNO, NETHERLANDS

## Abstract

**Background:**

Smoking among youths is a worldwide problem, particularly in China. Many endogenous and environmental factors influence smokers’ intentions to smoke; therefore, a comprehensive model is needed to understand the significance and relationship of predictors. This study aimed to develop a prediction model based on problem-behavior theory (PBT) to interpret intentions to smoke among Chinese youths.

**Methods:**

We conducted a cross-sectional study of 26,675 adolescents from junior, senior, and vocational high schools in Shanghai, China. Data on smoking status, smoking knowledge, attitude toward smoking, parents’ and peers’ smoking, and media exposure to smoking were collected from students. A structural equation model was used to assess the developed prediction model.

**Results:**

The experimental smoking rate and current smoking rate among the students were 11.0% and 3%, respectively. Our constructed model showed an acceptable fit to the data (comparative fit index = 0.987, root-mean-square error of approximation = 0.034). Intention to smoke was predicted by perceived environment (β = 0.455, *P* < 0.001) system consisting of peer smoking (β = 0.599, *P* < 0.001), parent smoking (β = 0.152, P < 0.001), and media exposure to smoking (β = 0.226, *P* < 0.001), and behavior system (β = 0.487, *P* < 0.001) consisting of tobacco experimentation (β = 0.663, *P* < 0.001) and current smoking (β = 0.755, *P* < 0.001). Smoking intention was irrelevant for personality system in students (β = -0.113, P>0.05) which consisted of acceptance of tobacco use (β = 0.668, *P* < 0.001) and academic performance (β = 0.171, *P* < 0.001).

**Conclusion:**

The PBT-based model we developed provides a good understanding of the predictors of intentions to smoke and it suggests future interventions among youths should focus on components in perceived environment and behavior systems, and take into account the moderating effects of personality system.

## Introduction

As the largest cigarette manufacturer and consumer in the world, China, according to national surveys conducted in 1984, 1996, 2002 and 2014, has also seen a substantial increase in the prevalence of cigarette smoking among adolescents [1–2]. There is an increase of approximate 80,000 to 90,000 new smokers among Chinese adolescents aged 12–17 years per day and nearly 11–12 million adolescents experimented with smoking in the past month [3].The statistics in 2011 shows that there are about 15 million current smokers and 40 million people who are trying to smoke among 130 million adolescents aged 13 to 18 in China [4]. More alarming is that the average age of smoking onset is decreasing [5]. Given the health-related consequences [6] resulting from tobacco use, preventing youths from smoking initiation remains a public health priority [7].

Intentions to smoke—thoughts developed before smoking behavior about experimenting with smoking [8]—are worth paying special attention to in adolescent smoking. Pierce’s longitudinal study pointed out that youths’ resolve to remain smoke-free against future smoking initiation is strongly associated with future smoking behaviors [9]. Several studies have also highlighted the function of intentions to smoke or not to smoke in predicting future smoking behaviors or serving as protection against future established smoking [10–14]. It has been reported that smoking intention among youths was the most powerful predictor of future smoking behavior [15].

A number of plausible theories and models could be used to explore related factors concerning smoking intentions and smoking behavior among youths, including the theories of reasoned action (TRA) and planned behavior (TPB) [16], information–motivation–behavioral [17]; and the attitude–social influence–efficacy [18] and transtheoretical stages of change models [19]. Personal and external determinants related to adolescent smoking intentions may include psychosocial, psychological, and behavioral factors. Psychosocial influences include: models from peers, parents, and siblings; parental monitoring and school intervention programs; tobacco marketing; and exposure to antismoking messages. Psychological and behavioral factors include: perceived instrumental values toward smoking; perceived behavioral control over smoking and self-efficacy; perceived normative beliefs about smoking; past smoking experience, and so on [20–27].

However, with the emergence of the argument proposed by some researchers [28] that smoking initiation among adolescents is unplanned behavior, theories such as the stage-like progression of smoking initiation and TRA and TBP have shown their limitations in addressing adolescent smoking because they rely too much on motivational and stage progression aspects to explain the initiation of risky behaviors. Meanwhile, the conceptualization of adolescent smoking [29] as more of a problem behavior than a health-compromising behavior provides a solid footstone for the application of problem-behavior theory (PBT), which is a social-psychological theory developed to account for proneness to engage in problem behaviors [30]. Therefore, the call for an integration of behavioral and motivational perspectives in dealing with tobacco use strongly facilitates the use of PBT, which incorporates both behavioral and psychosocial factors in its explanation of problem behaviors.

Based on the fundamental premise that behaviors result from person–environment interaction, PBT consists of three independent, but related systems: the personality system, which includes sociocognitive variables that are reflective of social leaning and developing processes such as the goal of academic achievements and attitudinal tolerance of deviance; the perceived environment system, which refers to role models, controls, support, influence, and expectations of others; and the behavior system, which consists of both problem and conventional behaviors [31]. The interplay of risk and protective factors within each system determines the overall likelihood of onset of problem behaviors. Also, the moderating effect of protective factors has been added to the recent reformulation of PBT [32]. Over the years, PBT has been applied in both developed and developing countries and proved useful in interpreting the etiology and maintenance of tobacco use among adolescents [33–35].

### Aim of the study

We reviewed worldwide research on the application of PBT in studying adolescent smoking and found that they rarely focused on smoking intentions and none had researched intentions to smoke among Chinese youths. The outcomes of those studies were usually confined to single factors that were loosely connected with each other in explaining smoking intentions. There was an absence of a more comprehensive network analysis, such as multiple-factor analysis, path analysis, and model construction, in applying smoking interventions practically. Even the South Korean model [25] developed to specifically predict future smoking intentions among adolescents has certain limitations in theoretical basis and the comprehensiveness of variable construction.

**Fig 1:**
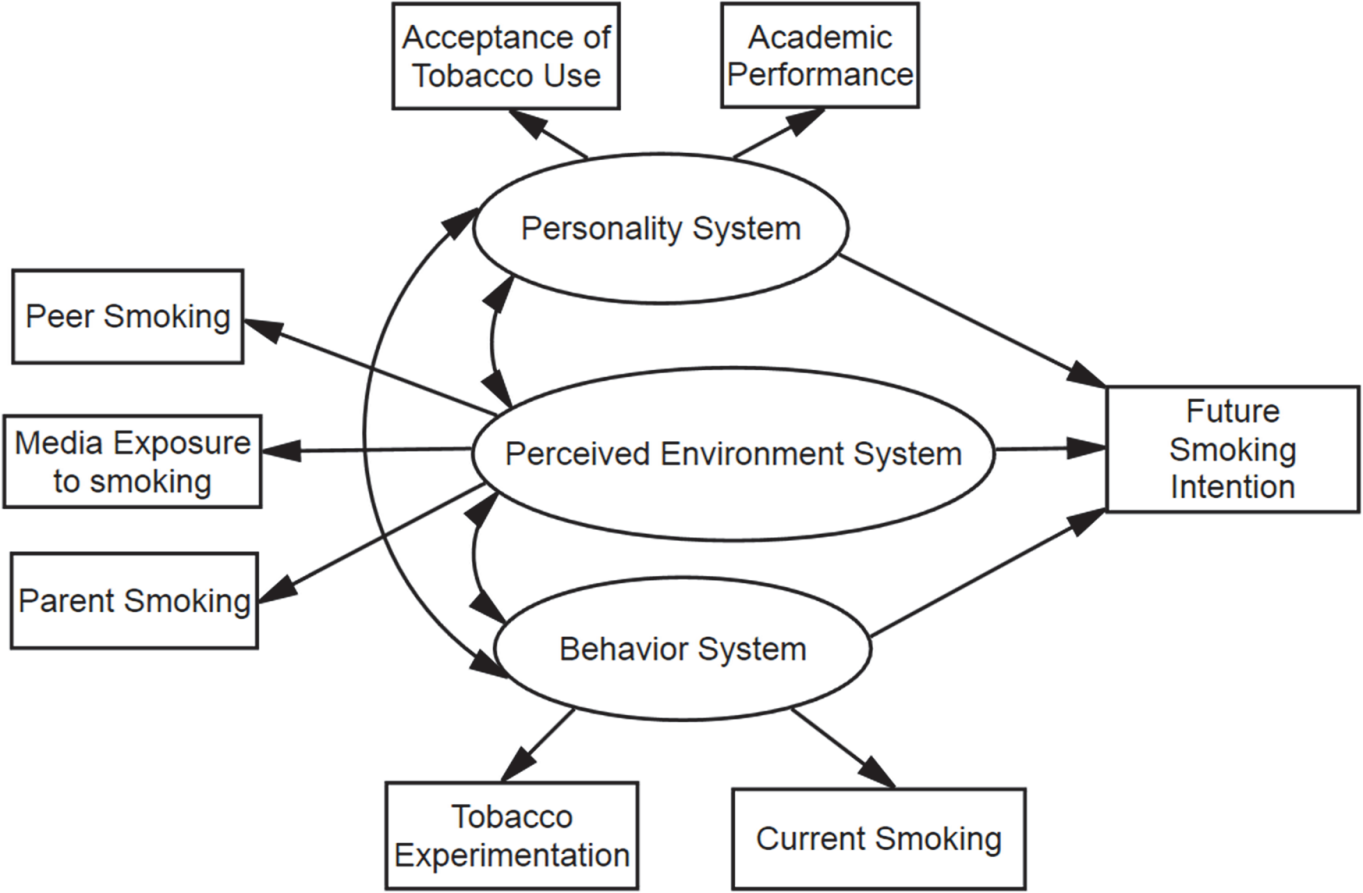
Hypothesized model for associations between problem behavior theory variables and adolescent future smoking intentions. Ovals represent multiple-indicator latent variables, rectangles represent single-indicator observable variables. Single-headed arrows represent regression path and double-headed arrows represent correlations.

In this study, we tried to explore determinants of future smoking intentions among youths using the framework of PBT and attempted to first develop a PBT-based prediction model (Fig. 1) to interpret the significance of some components in PBT systems and their internal relations in explaining the smoking intentions among youths in China. Focusing on adolescents’ intentions to smoke based on PBT, we hope to provide new directions and references for enhancement and optimization of tobacco control aimed at youths.

## Methods

### Ethics statement

The study was approved by the Ethics Committee of the School of Public Health, Shanghai Jiao Tong University. All school organizers, students, and their guardians provided written informed consent before enrollment in the study. The objectives, procedures, and potential risks and benefits of the study were all included in the informed consent.

### Study population and sampling

Shanghai is the largest city in China with a population of 23 million. A cross-sectional study was conducted among adolescents from junior, senior, and vocational high schools in Shanghai. All the data were obtained via a two-stage cluster sample by using a Chinese version of the Global Youth Tobacco Survey (GYTS), which had been tested in preliminary research and showed suitable reliability and validity [36–37]. The GYTS used a standardized methodology for constructing sampling frames, selecting participants, conducting field procedures, and processing data analysis [36]. In the first stage, five districts of Shanghai were randomly selected and all junior, senior, and vocational high schools in these districts were included as the sampling frame. Sixty-seven schools were randomly chosen from the districts determined as the subjects of study. In the second sampling stage, 871 classes within chosen schools were randomly selected and all students who attended school the day the survey was administered were eligible to participate. However, ninth-grade and twelfth-grade students were excluded in the sampling frame because they were busy preparing for the senior high school entrance examination or the national college entrance examination at the time of sampling. The sample size of the field survey was 27,308 students, from whom 26,675 usable questionnaires were collected for a response rate of 97.7%.

### Measure and data collection

The theoretical framework of PBT included three major systems of explanatory variables: the personality, perceived environment, and behavior systems. Each system was composed of several variables that could be observed and measured directly. Observable variables in each system served as instigations for engaging in problem behavior. Future smoking intentions acted as the dependent variable in the framework.

#### The Personality system

Two levels of variables constituted the personality system in this study. One level included the sociocognitive variables of values, expectations, beliefs, attitudes that reflected social learning, and developmental experience. The variables were drawn from a questionnaire on the acceptance of tobacco use in this study, which contained nine items (e.g., “Do you think smokers are more attractive?,” “Do you think smokers are more elegant?,” and “Do you think smokers are more popular?”). The answer to each item was assessed via a four-point Likert scale (1 = “disagree”; 4 = “agree”). Factor analysis showed equal coefficients for each of the nine items, which suggested that the sum of these items could form a composite scale as an index of the acceptance of tobacco use (Cronbach’s alpha coefficient = 0.743; range 9–36), where a higher score indicated a higher acceptance of tobacco use.

The second level of variables related to the proneness to problem behavior in the personality system, such as low academic achievements, lower self-esteem, or high value on independence. Numerous studies have revealed the importance of low academic achievements as a risk factor for problem behaviors [12,14–15]. In this study, students were asked to evaluate their academic achievements with “poor,” “average,” or “good” responses. Answers of “good,” “average,” and “poor” were scored as 1, 2, and 3, respectively. Higher scores indicated the lower level of the participants’ academic achievement at school.

#### The Perceived environment system

The concepts that constitute the perceived environment system include variables such as role models, social controls, and support. In this study, the perceived environment system was measured using three latent index variables.

The first index explored peer models of smoking as measured by the question, “How many of your four closest friends smoke cigarettes?” The responses were assessed by a four-point Likert scale (1 = no one; 2 = someone; 3 = most of them; 4 = everyone). A higher score indicated a strong peer effect on tobacco use.

The second index explored the parent smoking model as measured by the question, “Does your mother/father smoke cigarettes?” The responses were assessed by a three-point Likert scale (1 = both parents do not smoke; 2 = father or mother smokes; 3 both parents smoke). A high score indicated a strong parent smoking model.

The third index explored media exposure to smoking, which contained four items to assess exposure to tobacco-related advertisements and social activities among youths, e.g., “Have you seen tobacco-related advertisements on TV or at the movies during the past month?”; “Have you seen a tobacco-related advertisement in a newspaper during the past month?” The responses were assessed via a four-point Likert scale (1 = never; 4 = always). The sum of the scores of these four items was taken as an index of media exposure to smoking (Cronbach’s alpha coefficient = 0.702; range of 4–16), where a higher score indicated a higher level of media exposure to tobacco use.

#### The behavior system

The variables in the behavior system referred to prior experience with tobacco, including experimental and current smoking. The students were asked two questions, “Have you ever tried or experimented with cigarette smoking, even one or two puffs?” and “Have you used tobacco in the past 30 days?” The responses were assessed to form the two dichotomous variables (1 = “no”; 2 = “yes”).

#### Future smoking intentions

As the final outcome indicated by the model, future smoking intentions were assessed by three items: “Will you smoke if your best friend give you a cigarette”; “Will you use tobacco in the next 12 months?’, and ‘Will you use tobacco in the next 5 years?” Answers were assessed by a four-point Likert scale (1 = completely, no; 4 = completely, yes). The sum of the scores of the three items was taken as the index of future smoking intentions (Cronbach’s alpha coefficient = 0.78; range of 3–12). Higher scores indicated the participants were more likely to smoke in the future.

### Statistical analysis

The complex samples procedure of the Statistical Package for Social Sciences (version 20.0 for Windows; IBM SPSS Statistics, Armonk, NY, USA) was used to perform the descriptive statistical analysis including percentages, 95% confidence interval (CI), means (95% CI), and standard deviation (SD). A weighting factor was applied to each student record to adjust for nonresponses and variation in the probability of selection at the school and class levels. A stratified analysis was performed through binary logistic regression to detect the associations between socio-demographic variables and smoking experimentation and current smoking prevalence, which were indicated by ORu (univariate odds ratio). A structural equation model was used to test the theoretical model of future smoking intentions based on PBT with AMOS 20 (IBM SPSS). Model fit was assessed by the comparative fit index (CFI), the root-mean-square error of approximation (RMSEA), and the likelihood ratio by the chi-square test. The CFI ranges from 0 to 1 and a value greater than 0.9 indicates a good fit [38]. The RMSEA can range from 0 to infinity and it accounts for model complexity, and a value ≤ 0.05 indicates a close fit [39]. A non-significant likelihood ratio chi-square test suggests a good model fit, but the chi-square test is more sensitive to sample size than CFI and RMSEA [40]. A preliminary confirmatory factor analysis model was constructed to examine the factor structure and the relationships among all the variables based on PBT.

## Results

### Participants’ characteristics

A total of 26,675 students completed the survey, with an average age of 14.9 years (95% CI: 14.7–15.1 years; SD = 1.82 years; range 11–21 years). As shown in Table 1, 51.4% of the participants were male (N = 13,777, 95% CI: 50.4–52.4), 56.0% were from suburb areas (N = 14,871; 95% CI: 53.6–58.4). About half of the students were younger than 15 years old (55.0%; N = 14,618; 95% CI: 52.1–57.8) and 62.5% of the participants came from junior high schools (N = 16,519; 95% CI: 59.2–65.7). Eleven percent of the students were experimental smokers (N = 3096; CI: 10.3–11.8), but only 3.0% were current smokers (N = 871; 95% CI: 2.6–3.3). Most students (92.5%; N = 24,534; 95% CI: 91.9–93.0) considered themselves as consistent nonsmokers over the next 12 months, but 86.3% of the students responded they would not smoke over the next five years (N = 22,909; 95% CI: 82.5–89.1).

**Table 1:** Participant characteristics using the SPSS complex samples procedure (N = 26,675).

Characteristic variables	Number (%)	Weighting % (95% CI)
Gender		
Male	13,777 (51.6)	51.4 (50.4–52.4)
Female	12,898 (48.4)	48.6 (47.6–49.6)
Age (years)		
<15	14,618 (54.8)	55.0 (52.1–57.8)
15–17	7683 (28.8)	28.3 (26.2–30.6)
>17	4374 (16.4)	16.7 (14.8–18.8)
Hometown		
Urban	11,804 (44.3)	44.0 (41.6–46.4)
Suburb	14,871 (55.7)	56.0 (53.6–58.4)
School type		
Junior high school	16,519 (61.9)	62.5 (59.2–65.7)
Senior high school	5879 (22.0)	24.0 (21.0–27.3)
Vocational high school	4277 (16.0)	13.5 (12.1–15.0)
Academic performance		
Poor	7681 (28.8)	28.9 (28.2–29.6)
Average	11,640 (43.6)	43.7 (43.0–44.4)
Good	7354 (27.6)	27.4 (26.7–28.1)
Tobacco experimentation		
Yes	3096 (11.6)	11.0 (10.3–11.8)
No	23,579 (88.4)	89.0 (88.2–89.7)
Current smoking		
Yes	871 (3.3)	3.0 (2.6–3.3)
No	25,804 (96.7)	97.0 (96.7–97.4)
Smoking intention in the next 12 months		
Completely no	24,534 (92.9)	92.5 (91.9–93.0)
Maybe not	887 (3.4)	3.1 (2.8–3.3)
Maybe yes	893 (3.3)	3.2 (2.9–3.5)
Completely yes	361 (1.4)	1.2 (1.1–1.4)
Smoking intention in the next 5 years		
Completely no	22,909 (85.9)	86.3 (82.5–89.1)
Maybe not	1548 (5.8)	5.5 (5.1–5.8)
Maybe yes	1807 (6.8)	6.7 (6.3–7.2)
Completely yes	411 (1.5)	1.5 (1.3–1.7)

### Results for the stratified analysis by sex, age and school types

The associations between sex, age and school types and the prevalence for smoking experimentation and current smoking were presented in Table 2. The smoking experimentation rate for female and male students were 7.2% and 15.7% respectively (ORu = 2.39, 95%CI = 2.20–2.59, p<0.001), and the current smoking prevalence were 1.4% and 5.0% respectively (ORu = 3.73, 95%CI = 3.16–4.40, p<0.001). Students aged 15–17 reported higher prevalence for smoking experimentation (ORu = 2.98, 95%CI = 2.72–3.26, p<0.001) and current smoking status (ORu = 5.78, 95%CI = 4.78–7.00, p<0.001) than those aged below 15, and students aged above 17 demonstrated ever higher smoking experimentation (ORu = 4.10, 95%CI = 3.71–4.52, p<0.001) and current smoking prevalence (ORu = 7.64, 95%CI = 6.25–9.34, p<0.001), with the smoking experimentation rates for students aged below 15, 15–17 and above 17 to be 6.2%, 16.4% and 21.3% respectively, and the current smoking prevalence as 1%, 5.7% and 7.1% respectively. In terms of school types, compared with junior high school students, senior high school students reported higher smoking experimentation (ORu = 1.96, 95%CI = 1.77–2.16, p<0.001) and current smoking prevalence (ORu = 2.08, 95%CI = 1.65–2,61, p<0.001), and vocational high school students showed significantly higher prevalence for smoking experimentation (ORu = 5.69, 95%CI = 5.20–6.22, p<0.001) and current smoking (ORu = 13.92, 95%CI = 11.72–16.53, p<0.001). The smoking experimentation rate for the three groups were 6.8%, 12.4% and 29.2% respectively, and 1.1%, 2.2% and 13.2% respectively for current smoking prevalence.

**Table 2:** Socio-demographic factors associated with smoking experimentation and current smoking prevalence among the participants (N = 26675).

Smoking experimentation			Current smoking	
	N (row %)	ORu (95%CI)	N (row %)	ORu (95%CI)
Gender				
Female students	933(7.2)	1	180(1.4)	1
Male students	2163 (15.7)	2.39(2.20–2.59)*	691(5.0)	3.73(3.16–4.40)*
Age (years)				
<15	904 (6.2)	1	144(1.0)	1
15–17	1262(16.4)	2.98(2.72–3.26)*	418(5.7)	5.78(4.78–7.00)*
>17	930(21.3)	4.10(3.71–4.52)*	309(7.1)	7.64(6.25–9.34)*
School type				
Junior high school	1117 (6.8)	1	178 (1.1)	1
Senior high school	730(12.4)	1.96(1.77–2.16)*	130 (2.2)	2.08 (1.65–2.61)*
Vocational high school	1249(29.2)	5.69(5.20–6.22)*	563(13.2)	13.92(11.72–16.53)*

ORu: univariate odds ratio; 95% CI: 95% confidence interval.

* p<0.001.

### Validation of the theoretical model

**Fig 2:**
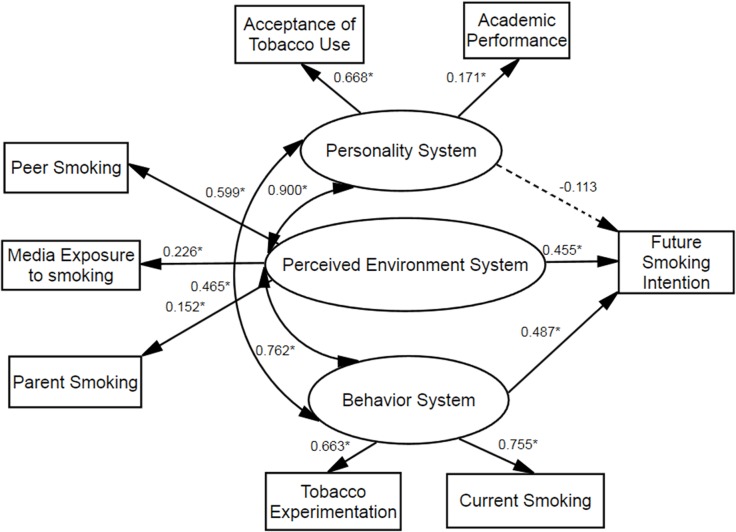
Final model for associations between problem behavior theory variables and adolescent future smoking intention. Ovals represent multiple-indicator latent variables, rectangles represent single-indicator observable variables. Single-headed arrows represent regression path and double-headed arrows represent correlations. Regression coefficients are standardized (*P < 0.001). Dotted line indicates non-significant path.

The summary statistics for each part of the theoretical model are shown in Table 3 and the model for the personality, perceived environment, and behavior systems related to future smoking intentions, including parameters and paths significance is shown in Fig. 2. The value of CFI was 0.987, which indicated a good fit and the RMSEA was within an acceptable range of 0.05 or less (RMSEA = 0.034; chi square = 474.19, degree of freedom = 15).

**Table 3:** Summary statistics of personality, perceived environment, and behavior systems related to future smoking intentions using the SPSS complex samples procedure (N = 26,675).

Scales	Mean (95% CI)	SD*
Personality system		
Acceptance of tobacco use (range 9–36)	15.08 (14.98–15.18)	3.99
Academic performance (range 1–3)	1.98 (1.97–2.00)	0.75
Perceived environment system		
Peer smoking (range 1–4)	1.59 (1.58–1.60)	0.57
Parents smoking (range 1–3)	1.35 (1.33–1.37)	0.54
Media exposure to smoking (range 4–16)	7.84 (7.79–7.88)	2.30
Behavior system		
Tobacco experimentation (range 1–2)	1.11 (1.10–1.12)	0.32
Current smoking (range 1–2)	1.03 (1.02–1.04)	0.18
Future smoking intention (range 3–12)	3.56(3.54–3.58)	1.53

* SD, standard deviation.

The model indicated that the perceived environment system (β = 0.455, *P* < 0.001) and the behavior system (β = 0.487, *P* < 0.001) were both predictors of intentions to smoke among youths. Furthermore, the model showed that all of the three systems in PBT were strongly interrelated: the personality and perceived environment systems (β = 0.900, *P* < 0.001), the personality and behavior systems (β = 0.465, *P* < 0.001), the perceived environment and behavior systems (β = 0.762, *P* < 0.001). The perceived environment system was predicted by peer smoking (β = 0.599, *P* < 0.001), parent smoking (β = 0.152, *P* < 0.001), and media exposure to smoking (β = 0.226, *P* < 0.001). The behavior system was predicted by tobacco experimentation (β = 0.663, *P* < 0.001) and current smoking (β = 0.755, *P* < 0.001). The personality system was not significantly related to future smoking intentions (β = –0.113, *P* > 0.05) and was predicted by acceptance of tobacco use (β = 0.668, *P* < 0.001) and academic performance (β = 0.171, *P* < 0.001).

## Discussion

In this study, 11.0% of the students from junior, senior, and vocational high schools in Shanghai experimented with smoking, which was lower than that in other countries. According to the 2000 GYTS reports, the experimental smoking rates ranged from 12.1% in Sri Lanka to 73.6% in Ukraine [41]. A study conducted in 2007 among 25,600 primary, high school and university students aged 8–24 in 32 regions of China showed that 23.1% had experimented with smoking [42]. An investigation among junior high school students in Hangzhou (a city locating very close to Shanghai) found the prevalence of tobacco experimentation was 9.7%, which was close to our results [43]. The current smoking rate among youths in our study was 3%, which was much lower than the 9.5% for the overall global rate in the GYTS 2000–2007 [44].According to the latest GYTS report [3] released by Chinese Center for Disease Control and Prevention in May 28th, 2014, the current smoking prevalence rate and the percentage for those trying to smoke among Chinese junior high school students were 6.9% and 19.9% respectively. Overall, according to the current study, the smoking prevalence among youths in Shanghai, China was relatively low compared with other developing countries and other regions in China, which may owe a lot to the implementation of “Action on Smoke-free School” in the past decade and the introduction of “Shanghai Regulations on Smoking in Public Places” in 2009. However, since the determinants of future smoking intentions may equally apply to other regions and given the large population and wide geographical variations and socioeconomic disparities of China and the increasing tendency among youths to smoke in five years as revealed in our study, there may be a large increase in the potential smoking population in the years to come.

The findings indicated that intentions to smoke among youths could be explained well by our model. In the study, the perceived environment system was strongly associated with future smoking intentions among youths and peer smoking largely defined intentions to smoke, which echoed numerous previous research studies. Scalici and Schulz pointed out that parents’ smoking and approval of smoking significantly contributed to increasing smoking intentions among adolescents [45]. Several studies have all indicated that peer influence was the strongest predictor of adolescents’ intentions to smoke, suggesting that peer smoking was related to less-negative attitudes towards tobacco use [46]. Media exposure, including cigarette advertising [47], newspaper coverage [48], magazine incidental smoking imagery [49], portrayals of smoking in movies, television, and so on [50], is believed to play a significant role in fueling youths’ intentions to smoke by shaping positive attitudes, beliefs, and expectations towards smoking, presenting attractive images, and inflating the perception of smoking prevalence. The volatile nature of the self means those adolescents vulnerable to social influences and their intentions to smoke will be shaped by environmental factors including family, peers, school, and media [8,51]. Therefore, an integrated prevention program that encompasses as many social and environmental factors as possible is warranted in tobacco control among youths.

The model also confirmed the function of the behavior system in influencing future smoking intentions among youths. This was in agreement with prior findings that both previous level of experimentation with cigarettes and the recency of these experiences strongly predicted future smoking [52–53]. A South Korean prediction model [25] also regarded past smoking experience as a determinant of future smoking intentions among adolescents. Therefore, those with previous tobacco use should be considered as a high-risk population when we design and implement tobacco prevention programs.

The most illuminating part of the current study is the clarification of the correlation among the three systems in PBT, which may give a comprehensive interpretation of the future smoking intentions among youths. All three components were correlated with one another. Although the personality system was not directly associated with smoking intentions, it showed a strong correlation (β = 0.900) with the perceived environment system, was closely related to the behavior system, and thus may be indirectly related to future smoking intentions. We propose that environmental factors may gradually influence elements in the personality system in some way; e.g., by fostering more positive attitudes and beliefs toward smoking, which could lead to more acceptance of tobacco use, or conversely, a higher level of acceptance of tobacco use may inflate youths’ perceived prevalence of smoking in their surroundings. An inclination to accept deviance like smoking may weaken students’ determination in the pursuit of academic success. It is understandable that previous tobacco use can promote the acceptance of tobacco use and frequent tobacco use can influence academic performances [54], or we can guess that students with higher acceptance of tobacco use and lower academic achievements are vulnerable to smoking. The function of environmental factors in determining smoking behavior has long been reported [55], and it has been found that overestimation of smoking prevalence was more common in experimental smokers and current smokers than in nonsmokers [56].

The model we constructed provided new insights into the design of more integrated and comprehensive smoking prevention. Efforts should be made to build a less or nonsmoking environment around youths, and much can be done to curb media promotion of smoking, such as banning, taxation, and policy requirements. In designing prevention programs, youths with prior smoking experience should be given priority and may differentiate from nonsmokers in terms of prevention strategy.

Several limitations should be acknowledged in the study. First, the selected participants came from the metropolis Shanghai, which restricts generalization of our results to rural areas. Second, ninth-grade and twelfth-grade students were excluded, so the results may not be reflective of all junior, senior, and vocational high school students. Third, our sampling covered a large age span, so a stratified analysis is necessary. Fourth, some variables in PBT such as self-esteem, conventional behaviors such as church attendance, and other problem behaviors such as problem drinking were not investigated. However, combining the national condition and considering that the main purpose of the study was to construct a PBT-based model to interpret smoking intentions among youths, some of these omissions could be forgiven. Fifth, the cross-sectional methodology does not permit causality, so that a longitudinal study design is required in the future.

However, to the best of our knowledge, our study is the first PBT-based model constructed to predict future smoking intentions among youths in China. The model identifies the contribution each system in PBT could make to predict intentions to smoke among youths and, more importantly, the associations among the variables. The findings in the study may serve as a baseline to design more integrated tobacco prevention programs aimed at young youths.
